# EF24 exerts cytotoxicity against NSCLC via inducing ROS accumulation

**DOI:** 10.1186/s12935-021-02240-z

**Published:** 2021-10-12

**Authors:** Minghui Chang, Ming Shang, Fang Yuan, Wei Guo, Cuijuan Wang

**Affiliations:** 1 Department of Clinical Laboratory, Shandong Cancer Hospital and Institute, Shandong First Medical University and Shandong Academy of Medical Sciences, Jinan, Shandong People’s Republic of China; 2 Department of Radiation Oncology, Shandong Cancer Hospital and Institute, Shandong First Medical University and Shandong Academy of Medical Sciences, Jinan, Shandong People’s Republic of China; 3 Ultrasound Diagnosis Department, Shandong Cancer Hospital and Institute, Shandong First Medical University and Shandong Academy of Medical Sciences, Jinan, 250117 Shandong People’s Republic of China; 4 Physical and Chemical Laboratory, Shandong Academy of Occupational Health and Occupational Medicine, Shandong First Medical University and Shandong Academy of Medical Sciences, Jinan, Shandong 250000 People’s Republic of China

**Keywords:** EF24, Reactive oxygen species, Non-small cell lung cancer

## Abstract

**Background:**

The role of Diphenyldifluoroketone (EF24), a synthetic analogue of curcumin with noteworthy antitumor potential, remains unclear in non-small cell lung cancer (NSCLC). Herein, the inhibitory effect of EF24 on NSCLC and its mechanism were studied.

**Methods:**

Cytotoxicity was measured by MTT assay, colony formation assay and xenograft model. Cell apoptosis and reactive oxygen species (ROS) level were quantified by flow cytometer. Protein level was detected by western blot assay. Mitochondria and autophagosomes were observed using transmission electron microscope and confocal microscopy.

**Results:**

In-vitro, EF24 significantly induced proliferation inhibition, apoptosis, mitochondrial fission and autophagy of NSCLC cell lines. These cytotoxic effects were significantly attenuated by two reactive oxygen species (ROS) scavengers, indicating its anti-cancer effects largely depend on ROS accumulation. In-vivo, EF24 inhibited tumor growth in a dose-dependent manner. Moreover, no pathological changes of heart, lung, spleen, kidney and liver of mice were observed. Collectively, EF24 induced ROS accumulation, in turn activates cell apoptosis, and then exerts its cytotoxicity on NSCLC cells.

**Conclusions:**

The results showed that EF24 exerted cytotoxicity against NSCLC via ROS accumulation. Thus, EF24 might serve as a potential anti-cancer agent for the treatment of NSCLC.

**Supplementary Information:**

The online version contains supplementary material available at 10.1186/s12935-021-02240-z.

## Introduction

Non-small cell lung cancer (NSCLC) is considered to be the leading type of cancers worldwide, accounting for about 80% of lung cancer cases, and its global morbidity and mortality have been showing a significant upward trend [1]. This is closely related to the negative impact of environmental and dietary factors, as well as the evolution of benign pulmonary diseases [2, 3]. NSCLC was viewed as a single disease entity before, and now subtyped as adenocarcinoma, squamous and large cell carcinoma. Adenocarcinoma accounts for 40% which is more likely to be detected before metastasis and in situ tends to have a better prognosis than other subtypes; Squamous carcinoma accounts for about 25% to 30%. It sometimes metastasizes later than other subtypes, which is highly sensitive to conventional treatment and has a better prognosis; Large cell carcinoma accounts for about 10% to 15%. Its rapid growth and spread make it harder to treat and has a poor prognosis [4, 5]. In recent years, although the treatment of NSCLC has been improved continuously, the improvement in survival rate is still not obvious. NSCLC still causes the most deaths, more than any other types of cancer [6, 7]. Among all treatments, chemotherapy is considered to be one of the most effective for NSCLC patients especially those at the advanced stage. Therefore, the development of high-efficiency as well as low-toxicity anti-cancer drugs has always been in demand.

Curcumin is a natural compound that has anti-inflammatory and antitumor effects. However, low bioavailability and efficacy hinder its further application [8]. To solve this problem, many new analogs were synthesized, among which EF24 is an excellent agent. EF24 shows enhanced bioavailability and more potent bioactivity, such as inhibiting the proliferation, movement and epithelial-mesenchymal transition of cancer cells [9, 10]. It also can induce cancer cell apoptosis and inhibit the metastasis of human tumor xenografts [10, 11]. However, the role of EF24 in NSCLC remains unclear.

ROS are responsible for maintaining redox homeostasis in cells. However, abnormally elevated ROS level can cause cell death in various ways [12]. Previous studies have shown that some chemotherapeutic drugs can increase ROS level in cancer cells and change the redox homeostasis [13–16]. Thus, enhancing intracellular ROS and disrupting the oxidative environment of cancer cells might be served as novel anti-cancer therapeutics. During normal ROS metabolism, the structure and shape of mitochondria maintain a dynamic balance between fission and fusion. However, excessive accumulation of ROS tends to break this dynamic balance and result in mitochondrial damage [17]. Mitochondrial damage can activate the cell to initiate the autophagy process, forming a double-membrane structure to wrap the damaged mitochondria and pass it to the lysosome [18]. Until now, the effect and mechanism of EF24 on regulating ROS of NSCLC cells remain largely unknown.

Herein, a series of molecular biology experiments were carried out to evaluate the potential effects of EF24 on NSCLC treatment and its anti-cancer mechanism. First, in-vitro assays such as MTT and colony formation evaluated the anti-cancer effects of EF24 on NSCLC cells. Rescue experiments confirmed the critical role of ROS accumulation. Further, in-vivo assay confirmed its effects on tumor growth, as well as evaluated the toxic effects in the main organs of nude mice. Our findings indicated that EF24 might exert an effective anti-cancer effect by increasing intracellular ROS, which provides a prospect for clinical NSCLC treatment.

## Materials and methods

### Pharmacological agents and antibodies

EF24 was purchased from Sigma-Aldrich (E8409-5 mg, US) and dissolved in Dimethyl Sulphoxide (DMSO, Sigma-Aldrich, D2650, Shanghai, China) to make stock solutions of 40 mM. ROS scavengers, Catalase (CAT, C1345) and N-acetyl-L-cysteine (NAC, A7250) were purchased from Sigma-Aldrich (Shanghai, China). Stock solutions of all drugs were stored at −20 °C. In all cases of cell treatment, the final DMSO concentration in the culture medium never exceeded 0.1%. Anti-bodies include LC3B (#3868), SQSTM1(#5141), Caspase3 (#2723), cleaved-Caspase3 (#9661), Cytochrome c (#11940), ACTB (#4970) and anti-rabbit IgG (#7074) were obtained from Cell Signaling Technology (Danvers, MA, USA). BAX (Cat No.50599-2-Ig) and COX-IV (Cat No.11242-1-AP) were bought from Proteintech Group (Wuhan, China).

### Cell culture

Human non-small cell lung cancer (NSCLC) derived cell lines A549, SPC-A1, H460 and H520 were purchased from American Type Culture Collection (Manassas, VA, USA) and China Center for Type Culture Collection (Wuhan, China). All these cells were cultured in DMEM (Gibco/Invitrogen, 11965084, USA) or RPMI 1640 (Gibco/Invitrogen, 31870082, USA) supplemented with 10% fetal bovine serum (Gibco/Invitrogen, 10100139C) and antibiotics (penicillin/streptomycin, 100 U/ml, 15070063, Gibco/Invitrogen) at 37 °C in 5% CO_2_.

### Cell viability assay

For cell growth/viability (MTT) assays, four cell lines were seeded in standard 96-well plates with 4000 cells per well and allowed to grow for 24 h to ensure attachment. The growth medium was then replaced with a medium that contained EF24 at the final concentration of 0 μM, 0.5 μM, 1 μM, 2 μM, 4 μM, 8 μM and 16 μM. After treatment for 24 h or 48 h, MTT assay was conducted through the addition of 3-(4,5-dimethylthiazol-2-yl)-2,5-diphenyltetrazolium bromide (Beyotime, ST316, Beijing, China) solution (made by adding 5 mg/mL in PBS) at 10 μL per well, followed by incubation at 37 °C in 5% CO_2_ for 4 h. Formazan crystals that formed were solubilized with 100 μL of acidified (0.01 M HCl) 10% SDS (sodium dodecyl sulfate). Bio-Rad 680 microplate reader (Bio-Rad 680, Bio-Rad Laboratories, Hercules, USA) was used to measure the absorbance at 570 nm.

### Colony formation assay

The growth ability of cells under EF24 treatment was evaluated by the colony formation assay. Depending on the cell line, 200 cells (A549, H460 and H520 cell lines) or 400 cells (SPC-A1 cell lines) were seeded into each well of a 6-well plate. EF24 was prepared for different concentrations (0, 1, 2, 4 μM) and added into the wells for 48 h, then the culture medium with EF24 were taken out and incubating with normal culture medium for 7–14 days. When the colony exceeded 50 cells, the cells were fixed with acetic acid–methanol (1:4) and stained with diluted crystal violet (1:30), and the number of colonies was counted. The colony formation efficiency was calculated with the following formula: Survival Fraction = Clones/Cell numbers × 100%.

### Apoptosis analysis

Apoptosis was evaluated by using the Annexin V-FITC Apoptosis Detection Kit (BD Biosciences Pharmingen, 556547, San Diego, USA) following the manufacturer’s instructions. In short, 1.5 × 10^5^ cancer cells of the four cell lines grown overnight in a 6-well plate were treated with the indicated concentration of EF24 (0, 1, 2, 4 μM) for 48 h. After that, the cells were stained with FITC-Annexin V and PI for 15–30 min in the dark, and then the fluorescence signal was detected using a FACS Calibur instrument (Becton Dickinson, Bedford, MA, USA). Data were analyzed using FlowJo Software 7.6 and three independent experiments were carried out.

### Measurement of ROS generation

Cellular ROS accumulation following treatment was measured using the DCFH-DA kit (Beyotime, S0033S, Beijing, China) according to the manufacturer’s protocol. Briefly, cells grown in 6-well plates were washed twice with serum free medium and subsequently incubated for 20 min with DCF-DA (20 μM, diluted in serum free DMED) at 37 °C. After the incubation and resuspension, fluorescence was measured by a FACS Calibur instrument (Becton Dickinson, USA) with the excitation source at 488 nm and emission at 525 nm.

### Analysis of mitochondrial morphology

Fluorescent immunocytochemistry (ICC) analysis of the mitochondrial network in A549 was carried out after treatment with different concentration of EF24. Cells were fixed in 4% paraformaldehyde (PFA) and permeabilized in 0.1% Triton X-100. After blocking with 3% BSA for 1 h at 25 °C, cells were incubated overnight with anti-COX-IV (1:200) at 4 °C, followed by incubation for 1 h at 25 °C with the appropriate fluorescently tagged secondary antibody. In this assay, 300 nM DAPI was used for nuclear staining (3 min at 25 °C in the dark). Finally, the mitochondrial morphology was observed on a confocal laser microscope (LSM800, Carle Zeiss, Germany).

### Western blots

First, 10^5^ cells per well were seeded into a 6-well plate. EF24 was prepared for different concentrations (0, 1, 2, 4 μM) and treated the cells for 48 h. Then, the cells were lysed with a lysis buffer (Beyotime, P0013J, Shanghai, China) added with a protease inhibitor (Beyotime, P1005, Shanghai, China). After centrifuging at 4 °C for 15 min, the protein concentrations were detected by a BCA kit (Thermo Fisher Scientific, 23225, Waltham, MA, USA). The subsequent western blot analysis was carried out in accordance with the routine procedure [19]. The primary antibodies dilution as follows: Cyto C (1:1000), BAX (1:1000) LC3B (1:1000), SQSTM1 (1:1000), Caspase3 (1:1000), cleaved-Caspase3 (1:500) and ACTB (1:2000) Anti-rabbit IgG was diluted at a ratio of 1:2000. Primary antibody was incubated overnight and secondary antibody was incubated for 1 h. The results were visualized using ECL substrate reagent kit (Thermo Fisher Scientific, 32209, Waltham, MA, USA) or detected by exposure to a film.

### Transmission electron microscope (TEM)

First, A549 and H520 cells were treated with control or EF24 (2 μM, 4 μM). Next, cells were fixed with 2.5% glutaraldehyde in 0.2 M HEPES overnight at 4 °C. The cells were then post-fixed in 1% OsO4 at room temperature for 60 min, stained with 1% uranyl acetate, dehydrated through graded acetone solutions, and embedded in polyed 812 resin (90529-77-4, SPI). Areas containing cells were block-mounted and cut into 70 nm sections and examined using an electron microscope (HT7700, HITACHI).

### *In-vivo* xenograft model

Four to six weeks-old female BALB/c nude mice were purchased from Beijing Huafukang Bioscience Co. Ltd. (Beijing, China). Mice were housed and handled in laminar flow cabinets under specific pathogen-free conditions with temperature at 25 °C ± 2 °C and a relative humidity of 70% ± 5% according to institutional guidelines and experimental procedures approved by the Institutional Animal Care and Use Committee of Shandong Cancer Hospital affiliated Shandong First Medical University. For constructing the model, 5 × 10^6^ A549 cells suspending in 100 μl PBS was injected into the left flank of nude mice. When the tumors reached approximately 100 mm^3^ in size, mice were randomly divided into four groups, and treated at the indicated dose [20] (EF24 5 mg/kg, 10 mg/kg or 20 mg/kg) intraperitoneally once a day for 17 days. The control group was treated with a solution containing DMSO. All animals were examined daily for general signs of distress and complications. The volume of local tumors was calculated by measuring two perpendicular diameters (length and width) every two days using a caliper. The mouse body weight was measured every two days using an electronic scale. The volume was calculated following the formula: tumor volume (mm^3^) = 1/2 × (length × square width). On the 17th day, mice were sacrificed according to the 2020 AVMA Guidelines on Euthanasia state. In short, the mice were anesthetized by intraperitoneal injection of 0.1 mL of 1% phenobarbital sodium, and then the spinal cord was disconnected from the brain with force and speed. After that, tumors were dissected and weighted.

### Immunohistochemistry and H&E staining

After the mice were sacrificed, their tumors, hearts, livers, spleens, lungs and kidneys were resected and immediately fixed in 10% formalin. The slides were sequentially subjected to the steps of dewaxing, hematoxylin staining, eosin staining, washing, dehydration, and fixing. Then, the specimen goes through the following procedures: antigen incubation, blocking, goat serum pre-incubation, hematoxylin–eosin staining, and incubation with, anti-cleaved-Caspase3 (1:500, GB11532, Servicebio Technology) or anti-Ki-67 (1:500, GB111141, Servicebio Technology). Secondary staining was carried out with HRP-conjugated anti-rabbit IgG and DAB peroxidase substrate. The IHC kit was purchased from ZSGB-BIO (PV-9000).

### Statistical analysis

Statistical significance was evaluated with data from at least three independent experiments. GraphPad Prism 6.02 (GraphPad Software, San Diego, CA, USA) was used for data analysis. Statistical analysis was carried out using student *t*-test. Data are presented as the mean ± SD. For all statistical tests, significance was established at *P* < 0.05.

## Results

### EF24 inhibits the growth and colony formation of NSCLC cells *in-vitro*

**Fig. 1:**
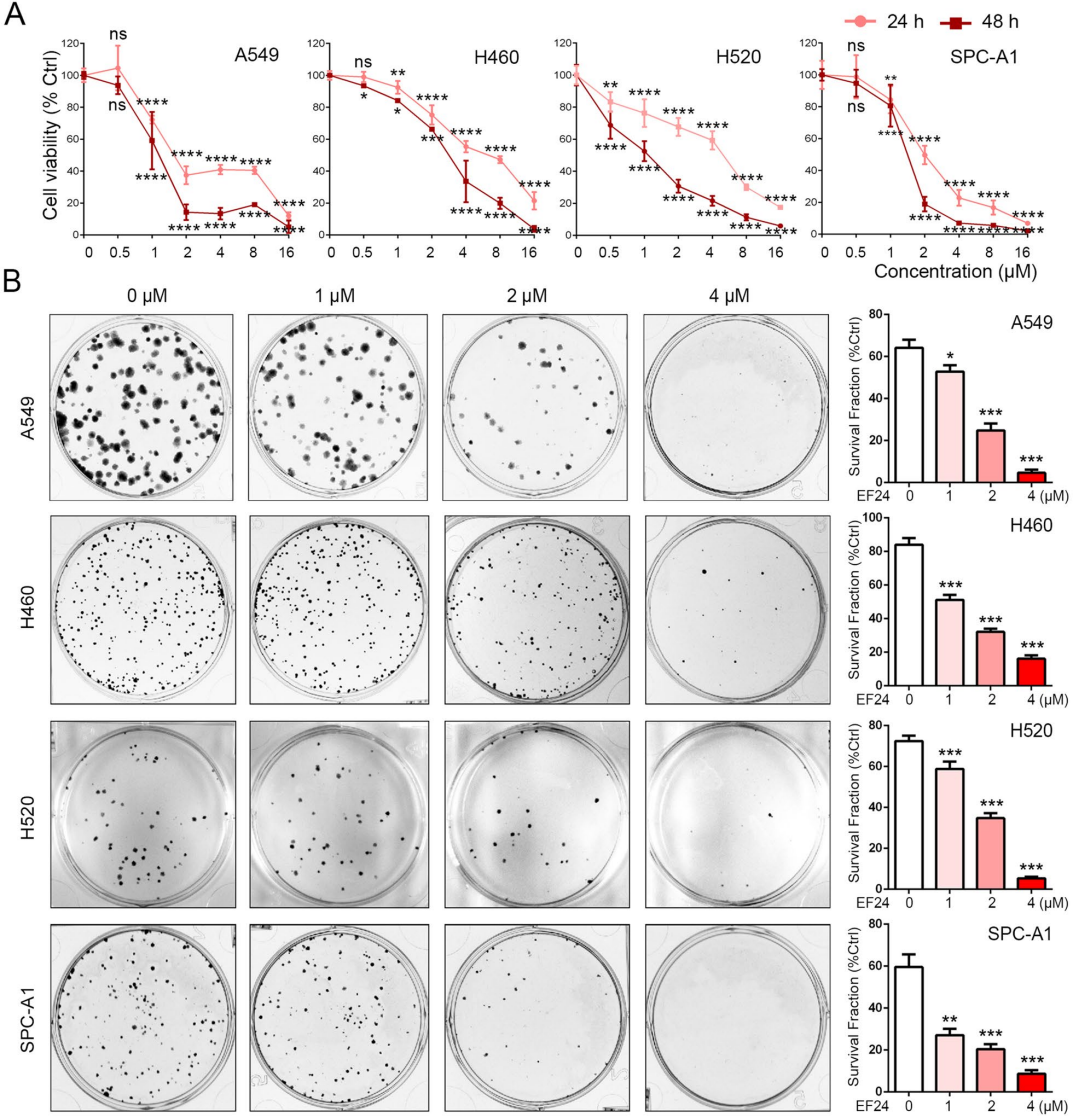
EF24 inhibits the growth of NSCLC cells in-vitro. **A** A549, SPC-A1, H460 and H520 cells were treated with the indicated concentrations of EF24 for 24 h and 48 h, and then subjected to MTT assay. The absorbance value was calculated and standardized to the control group. **B** The cells were treated with 0 μM, 1 μM, 2 μM and 4 μM EF24 respectively for 2 h and subjected to the cell colony formation assay. Surviving fraction is presented as mean ± SD, *P < 0.05, **P < 0.01, ***P < 0.01

To verify the cytotoxic potency of EF24 against NSCLC, we first determined the effects of EF24 on cell proliferation in four NSCLC cell lines (A549, SPC-A1, H460 and H520). All cells were exposed to the treatment with control or indicated concentrations of EF24 (1 µM, 2 µM and 4 µM), and subjected to MTT assay, respectively. As shown in Fig. 1A, EF24 significantly inhibited NSCLC cells viability in a dose-dependent manner. In addition, colony formation assay was also performed. As shown in Fig. 1B, colony formation capability of the above four NSCLC cell lines was significantly inhibited when treated with EF24. Taken together, these data supported the inhibitory role of EF24 in NSCLC cell growth and colony formation.

### EF24 inhibits tumor growth in-vivo

**Fig. 2:**
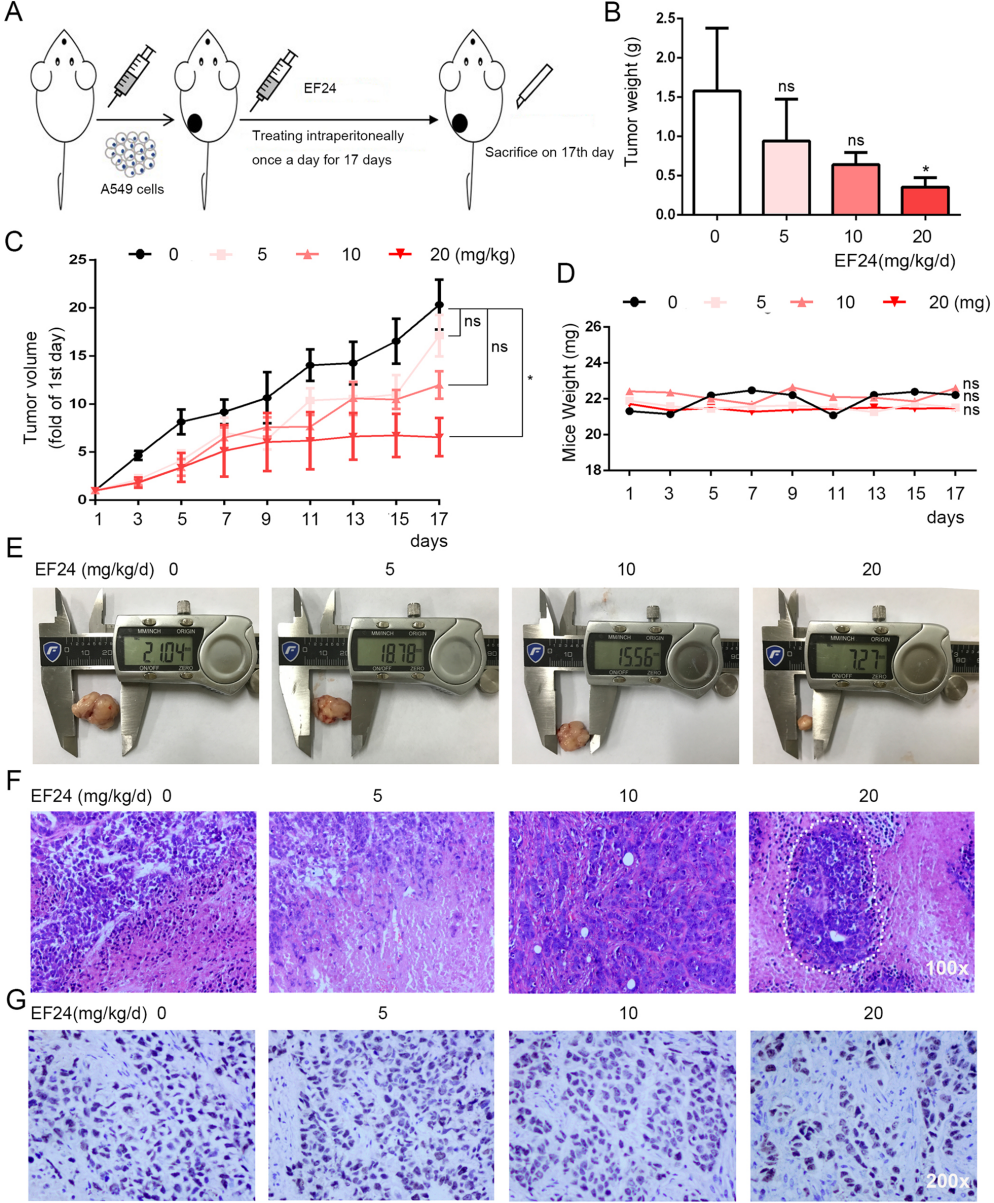
EF24 inhibits tumor growth in-vivo. **A** A brief flowchart of the experiment design in-vivo. A549 xenograft tumor was established and divided into the following four groups and received 17-day treatment: control, EF24 (5 mg/kg/d, 10 mg/kg/d and 20 mg/kg/d). **B** The weight of tumors. **C** The curves of tumor growth. **D** The body weight of mice. **E** Representative images of xenografts from different groups. **F** H&E staining tumor tissue sections of different groups. Magnification: 100×. **G** Anti-Ki-67 staining of tumor tissues in different groups. Magnification: 200×. The results shown are means ± SD; *P < 0.05

Furthermore, A549 NSCLC xenografts model was constructed to verify the antitumor effects (Fig. 2A). EF24 was used to treat the constructed tumor-bearing murine and the specific process was described in the material method section. As shown in Fig. 2B–E, a clear reduction trend was observed in both tumor volume and tumor weight after EF24 treatment, indicating the inhibitory effect of EF24 on murine xenograft tumors. After the sacrifice of the murine, the tumors and major organs were resected and subjected to HE or IHC staining. As shown in Fig. 2F, all xenograft had been constructed successfully, and necrosis lesion was observed in 20 mg/kg/d EF24 treated group but not in other treated groups. Due to expression of the Ki67 protein is related to the proliferative activity of intrinsic cell populations in cancerous tumors, IHC for Ki-67 was carried out to determine the expression level. The results revealed that EF24 (20 mg/kg/d) led to a significant decrease of Ki67 expression compared with control group (Fig. 2G). These results indicated that EF24 could inhibit NSCLC tumor growth and might induce tumor necrosis in-vivo.

**Fig. 3:**
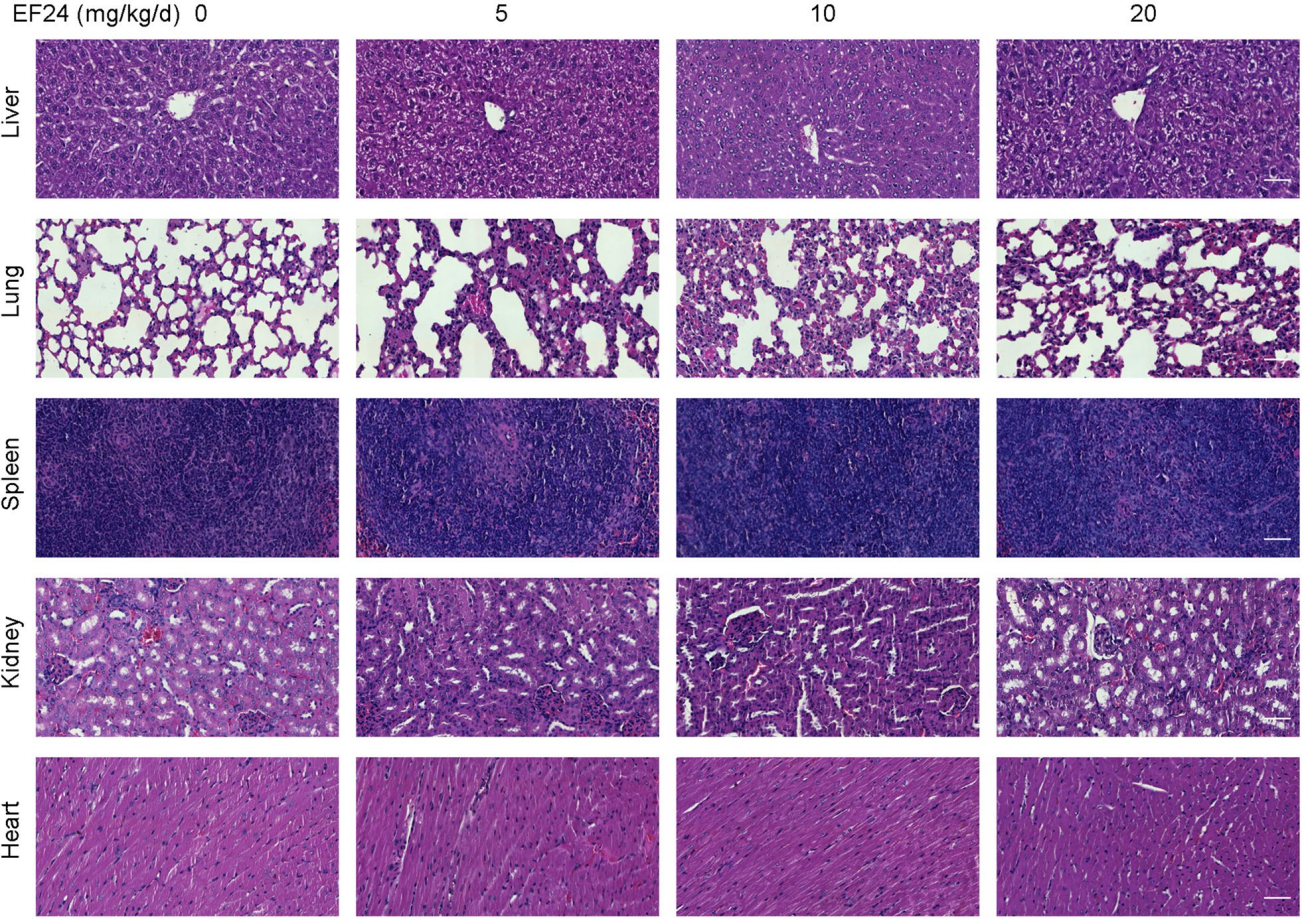
Histopathological analyses of major organs. Histopathological analyses of liver, lung, spleen, kidney and heart tissues of A549 xenograft. Scale bars: 50 μm

Naturally, the side effects and toxicity of EF24 were also assessed in tumor-bearing murine. As shown in Fig. 2D, no significant difference was found in the body weight of the mice treated with EF24 compared to the control group. To evaluate whether EF24 has obvious side effects, HE staining was performed on the main organs of mice. As shown in Fig. 3, the cell morphological and histological were normal in heart tissues, liver tissues, spleen tissues, lung tissues and kidney tissues of murine after EF24 treatment. No histopathological changes and abnormal characteristic cells were found. These results implied that EF24 might exert significant toxic effect on tumor tissue, but has no obvious toxicity on normal organs.

### EF24 induces apoptosis in NSCLC cells

**Fig. 4:**
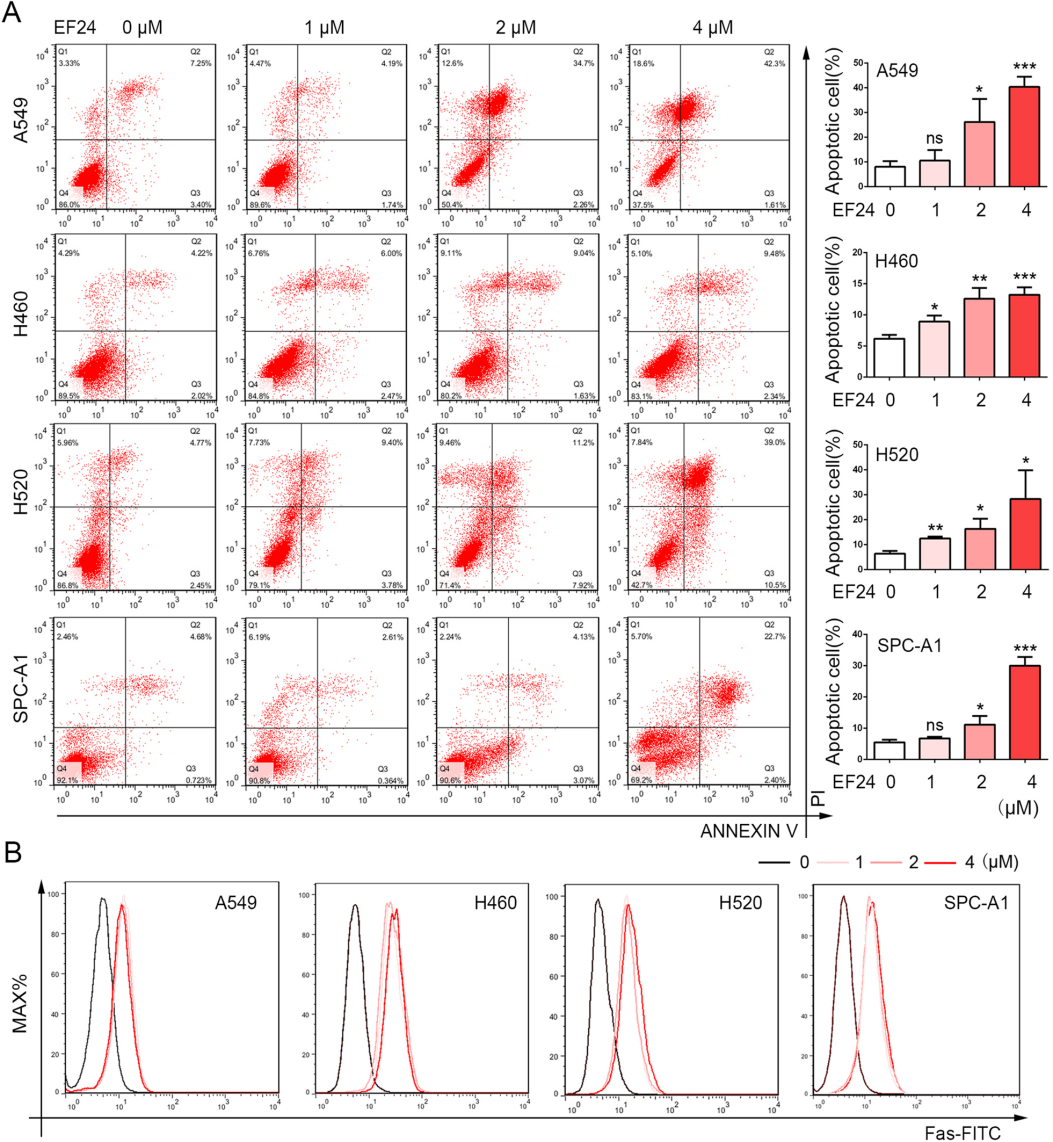
EF24 induces apoptosis in NSCLC cells. **A** A549, SPC-A1, H460 and H520 cells were treated with or without indicated concentrations of EF24 respectively for 48 h and subjected to apoptosis assay using Annexin-V & PI staining. **B** The expression of apoptotic marker Fas in cells were determined. Three independent experiments were performed and the results statistically analyzed as means ± SD, *P < 0.05, **P < 0.01, ***P < 0.01

To further investigate the underlying effects involved in EF24 treatment, apoptosis detection assay was performed in the four NSCLC cell lines. Flow cytometry analysis demonstrated that EF24 induced more apoptotic or dead cells in NSCLC cells (Fig. 4A). Previous study has proved that FAS is a transmembrane glycoprotein that functions as a cell death receptor of the TNFR (tumor necrosis factor receptor) superfamily, participating in the process of apoptosis [21]. Here, Fas-FITC assay was conducted by flow cytometry and the result demonstrated that EF24 induced increased apoptosis in NSCLC cells (Fig. 4B). Besides, western blot experiments were also performed to detected several vital apoptosis-related proteins after EF24 treatment in A549 and H520 cell lines. As shown in Additional file 1: Fig. S1A, EF24 induced the upregulation of cleaved-caspase3, BAX and cytochrome C, three important signals of mitochondrial apoptosis pathway. For further confirmation, the expression of cleaved-caspase3 was detected by IHC experiment (Additional file 2: Fig. S2B), which also confirmed that EF24 can induce more apoptosis *in-vivo*. All these results indicated that EF24 induced tumor cell apoptosis, which was an important anti-cancer mechanism.

### EF24 induces ROS production, mitochondrial fission and autophagy

**Fig. 5:**
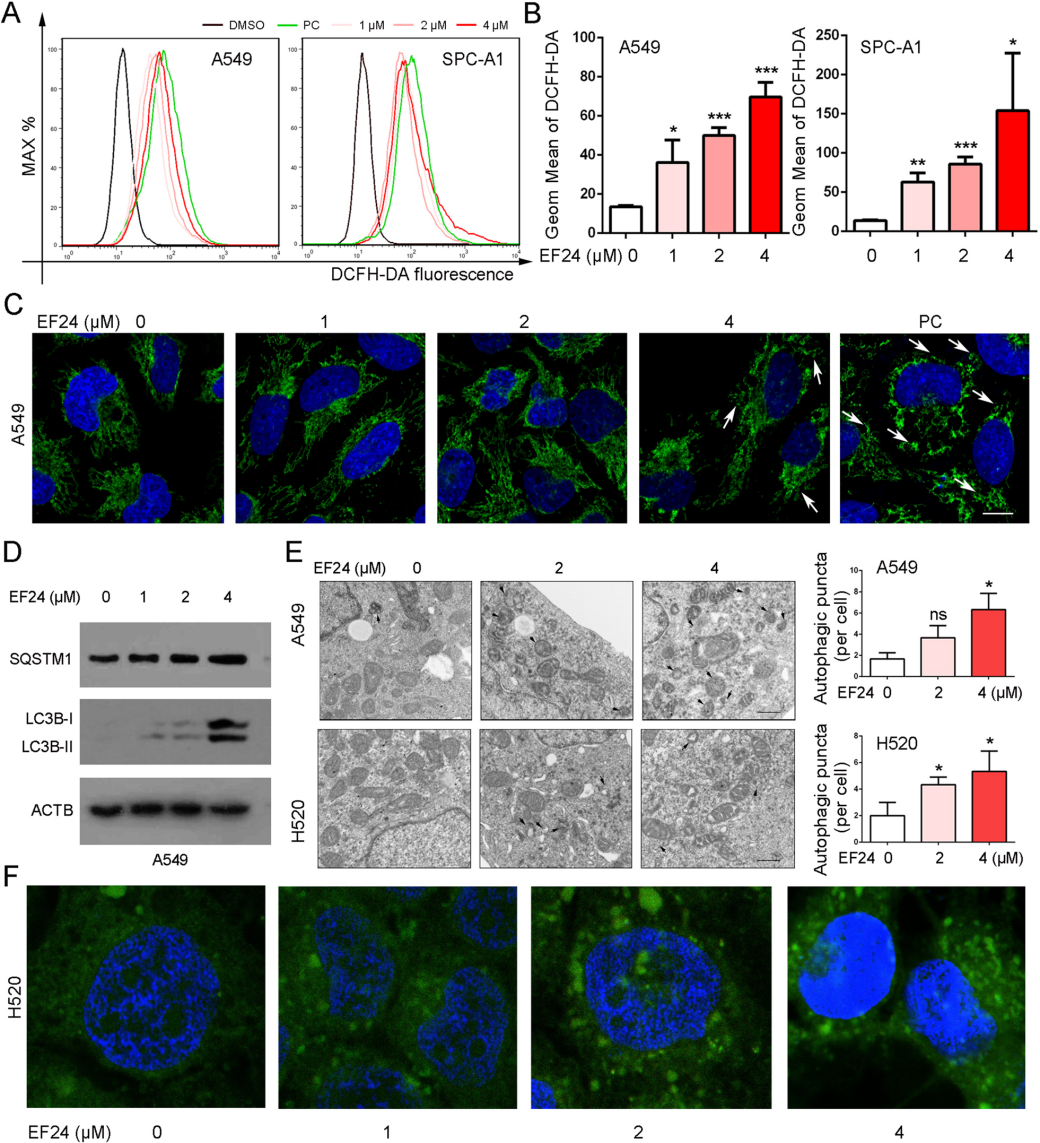
EF24 induces ROS accumulation, mitochondrial morphological changes and autophagy. **A**–**B** EF24 induces ROS accumulation. **C** EF24 induced mitochondrial fragmentation of the mitochondrial network in A549 cells. The arrow indicates fragmented mitochondria. **D** Western blot analysis demonstrated SQSTM1, LC3B and ACTB expression in EF24-treated A549 cells. **E** Autophagic vacuoles in A549 cells treated with various concentrations of EF24 were observed by TEM. The arrow indicates autophagic vacuoles. Number of autophagic vacuoles were calculated. **F** Changes in the localization of LC3B in cells assessed by immunofluorescence under confocal laser microscopy. The results are presented as mean ± SD, *P < 0.05, **P < 0.01, ***P < 0.01

ROS accumulation triggered by drugs is one of the main mechanisms of cell cytotoxicity [22]. DCFH-DA, a fluorescent probe, was used to analyze the potential effect of EF24 on ROS generation. As depicted in Fig. 5A and B, EF24 causes a dose-dependent increase in DCF-responsive ROS in NSCLC cells. It has been reported that ROS overproduction can activate mitochondrial morphological changes, which possesses an irreplaceable position in the regulation of cell death and apoptosis [23]. Herein, after treatment with EF24, the mitochondrial morphology in A549 cells was also determined by fluorescence immunocytochemistry. COX-IV, a protein localized on the inner mitochondrial membrane, was used to determine the morphology of mitochondria. As shown in Fig. 5C, EF24 treatment resulted in the accumulation of fragmented mitochondria with a shorter length and a smaller number of branches, indicating that the mitochondria underwent an imbalance of fusion and fission. This type of mitochondrial damage could initiate autophagy to removal dysfunctional mitochondria. SQSTM1 is a selective autophagic adaptor that can be incorporated with LC3B (an important marker of autophagy) and then be degraded by lysosomal hydrolyses [24]. As shown in Fig. 5D, autophagy of NSCLC cells was significantly activated by EF24, which was evidenced from the increased levels of LC3B-II and SQSTM1. For further confirmation, TEM detection assay was performed. Figure 5E showed that EF24 treatment significantly increased intracellular autophagic vacuoles, which appeared as double-membrane vesicles with visible cytoplasmic content. Fluorescence immunocytochemistry was also performed to detect the formation of autophagosomes by staining endogenous LC3B. Figure 5F showed that, compared with the control group, the fluorescence intensity of the H520 cells treated with EF24 showed more autophagy. Taken together, these observations supported that EF24 can induce ROS accumulation, mitochondrial morphological changes and autophagy.

### EF24 induces cytotoxicity is blocked by CAT and NAC

**Fig. 6:**
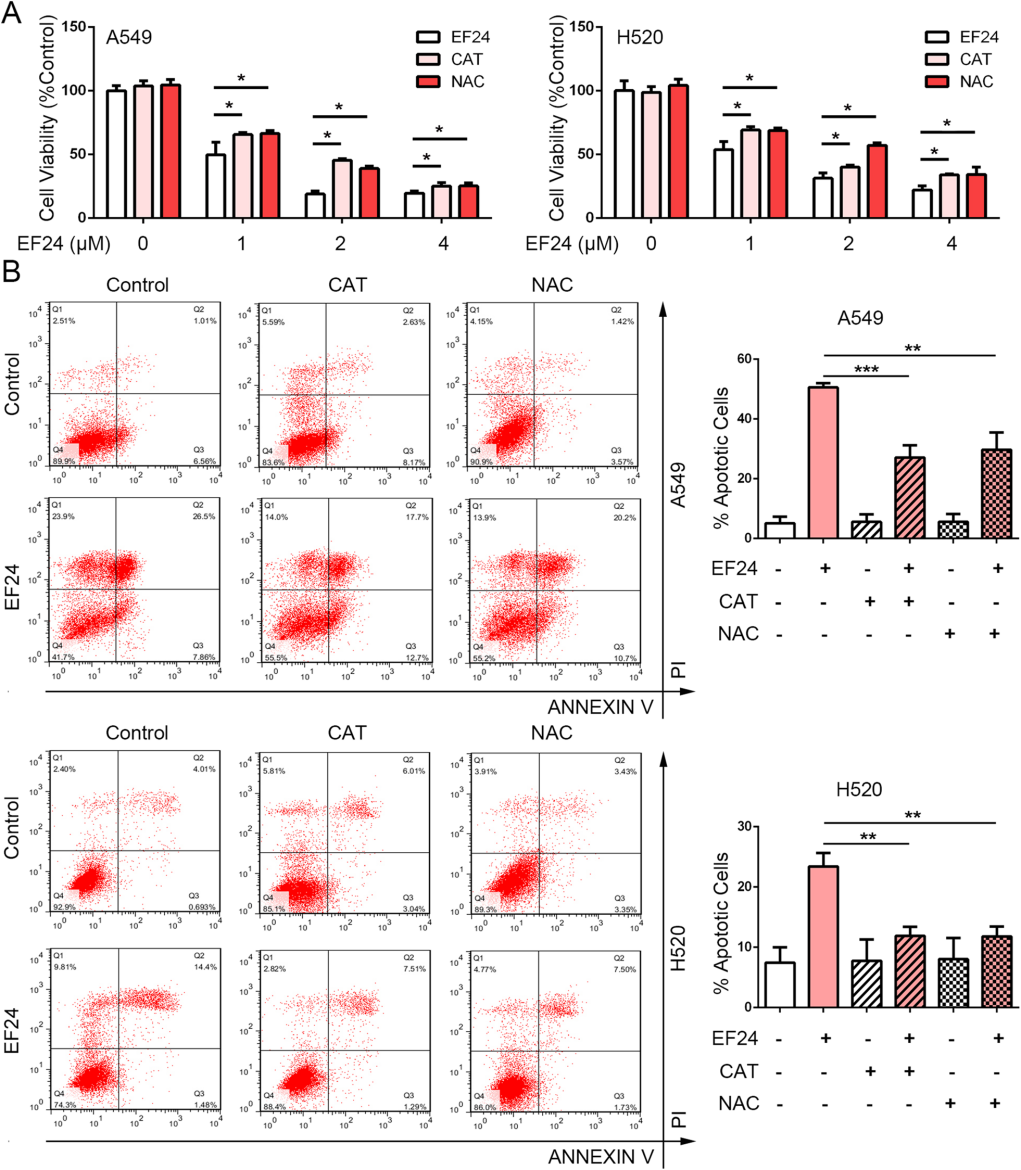
EF24 induces cytotoxicity is restored by ROS scavengers. A549 and H520 cells were treated with 4 μM EF24 for 48 h with or without CAT and NAC employed 2 h before EF24 treatment, after that cell viability using MTT assay (**A**) and apoptotic cells using Annexin-V & PI staining (**B**) were detected. Three independent experiments were performed and the results statistically analyzed as means ± SD, *P < 0.05, **P < 0.01, ***P < 0.01

To determine the role of ROS accumulation in the anti-cancer effects of EF24, a rescue experimental strategy was performed via scavenging ROS by catalase (CAT) and N-acetyl-L-cysteine (NAC), two ROS scavengers. And then MTT assays and Flow cytometry were conducted to detected the cell proliferation and apoptosis in different drug treatment groups. As shown in Fig. 6A and B, compared to the group with EF24 treatment alone, the proliferation ability of cells in the CAT or NAC pretreatment group was significantly restored, and the proportion of apoptotic cells was significantly reduced. These results proved that after efficiently blocking the ROS accumulation, the cytotoxicity of EF24 in NSCLC cells was significantly reduced. These illustrated that EF24 exhibits cytotoxicity mainly depend on ROS accumulation.

## Discussion

In the present study, EF24 was demonstrated to exert anti-cancer activity in-vitro and in-vivo mainly via inducing ROS generation coupled with mitochondrial morphological changes and autophagy, showing great anti-cancer potency in NSCLC.

ROS are considered to be second messengers and are involved in physiological processes such as apoptosis, proliferation and cancer progression [25]. However, excessive ROS tends to cause damage to cells. It has been proved that high levels of ROS can induce various types of cell death and therefore exert tumor suppressor effects [12]. In addition, it has been proven that many effective cancer chemotherapy drugs can induce high levels of oxidative stress in tumor cells [26]. Therefore, the utilization of ROS accumulation for cancer treatment has great potential. Mitochondria are main sites for the generation and release of ROS [27]. In turn, ROS can also cause mitochondrial dysfunction and mitochondrial-mediated apoptosis [28]. Although it is known that curcumin exhibits high anti-cancer activity by generating ROS [29–31], there is insufficient research on the anti-cancer effect of its analogue EF24 and its mechanism. Therefore, in order to obtain more information about EF24, the ROS level and mitochondrial morphology in EF24-treated cells were measured in this study. The results suggested that EF24 induced accumulation of ROS and obvious mitochondrial fission in NSCLC cells. Compared with curcumin, EF24 induced cancer cell apoptosis and inhibit the growth of human tumor xenografts at a much lower dose [32,33]. In animal experiments, the dosage of EF24 that inhibit tumor growth is generally only one tenth of curcumin [34]. From the previous literature and our research, among curcumin analogs, EF24 was shown to be a good representative of excellent inhibition of cancer cells [35].

In the past 10 years, the molecular mechanism and network of action of autophagy have been realized. Although autophagy is involved in different cellular biological activities due to substrate and receptor specificity, cellular redox status still possesses a key role in the whole process of autophagy. Recent developments have clarified the vital position of ROS in the process of autophagy [36, 37] and the molecular regulation mechanism between ROS and autophagy has gradually been elucidated [38,39]. Since EF24 induced an increase in intracellular ROS levels, here we speculated that EF24 may induce autophagy in cells and verified the expression of LC3B, SQSTM1 in NSCLC cells after treated with EF24. Our results proved that EF24 could induce expression of autophagy-related proteins and formation of autophagosomes.

To further determine the effect of ROS on the anti-cancer activity of EF24, the antioxidants was applied in this study. Two ROS scavengers (CAT and NAC) were used to evaluate the changes in the anti-cancer effect of partial removal of ROS. The results showed that the cytotoxicity induced by EF24 was effectively attenuated by these two ROS scavengers, as evidence of restoration of cell viability and apoptosis. Taken together, the current study supported that EF24 exhibit cytotoxicity via ROS accumulation.

Several limitations should be carefully considered in the present study. Although the discovery that EF24 plays an anti-cancer role by inducing ROS accumulation is very interesting, the underlying molecular mechanisms are unknown. We hypothesized that EF24 might act on mitochondria, thus affecting the normal metabolism of ROS in NSCLC cells. In addition, more evidence, such as pharmacokinetic trials, is still lacking for EF24 to be tested in clinical trials in the future. More importantly, the employment of EF24 as a therapeutic sensitizer might be more promising in the era when combination therapy strategies are used in cancer treatment. Absolutely that also needs more efforts and will be the direction of our future work.

## Conclusion

This study has demonstrated that EF24, an analog of curcumin, exhibited excellent cytotoxicity on NSCLC cells by inducing ROS generation and subsequent mitochondrial dysfunction, autophagy and apoptosis. In-vivo, EF24 also showed significant inhibition of tumor growth. What's more, EF24 induced anti-cancer effects at a much lower dose than curcumin, and no significant toxic effects on tumor-bearing mice was observed. Therefore, EF24 might serve as a potential agent for the treatment of NSCLC patients. The ROS-mediated anti-cancer perspective also provides a new strategy for the design and development of anti-cancer drugs.

## Supplementary Information

**Additional file 1: Fig. S1.** EF24 induces apoptosis in-vitro and in-vivo. (A) After treating A549 and H520 cells as above indicated, western blot assays were performed using antibodies with Caspase3, cleaved-Caspase3, BAX, Cytochrome C and ACTB. (B) After the mice were sacrificed, tumors were performed IHC staining using antibody cleaved-Caspase3. Scale bars: 20 μm.

**Additional file 2: Fig. S2.** Schematic Illustration of the main research methodology.

## Data Availability

Please contact author for data requests.
