# Supplementary figures and images for: EF24 exerts cytotoxicity against NSCLC via inducing ROS accumulation
Source: Cancer Cell Int. 2021 Oct 12;21:531. doi: 10.1186/s12935-021-02240-z (PMC8513219; doi:10.1186/s12935-021-02240-z)

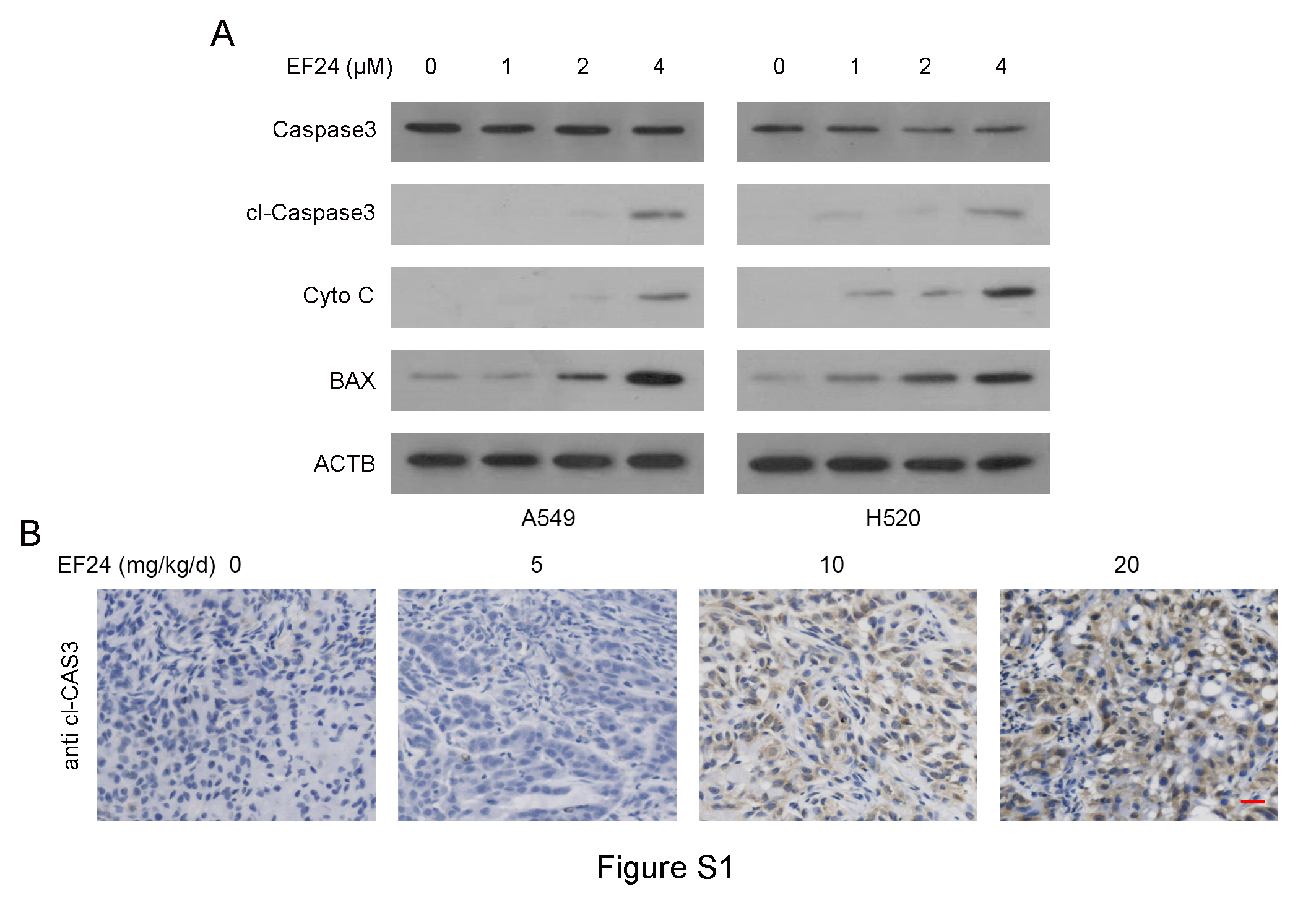

Supplement: Supplementary file 1 — Additional file 1: Fig. S1. EF24 induces apoptosis in-vitro and in-vivo. (A) After treating A549 and H520 cells as above indicated, western blot assays were performed using antibodies with Caspase3, cleaved-Caspase3, BAX, Cytochrome C and ACTB. (B) After the mice were sacrificed, tumors were performed IHC staining using antibody cleaved-Caspase3. Scale bars: 20 μm. [file 12935_2021_2240_MOESM1_ESM.jpg]

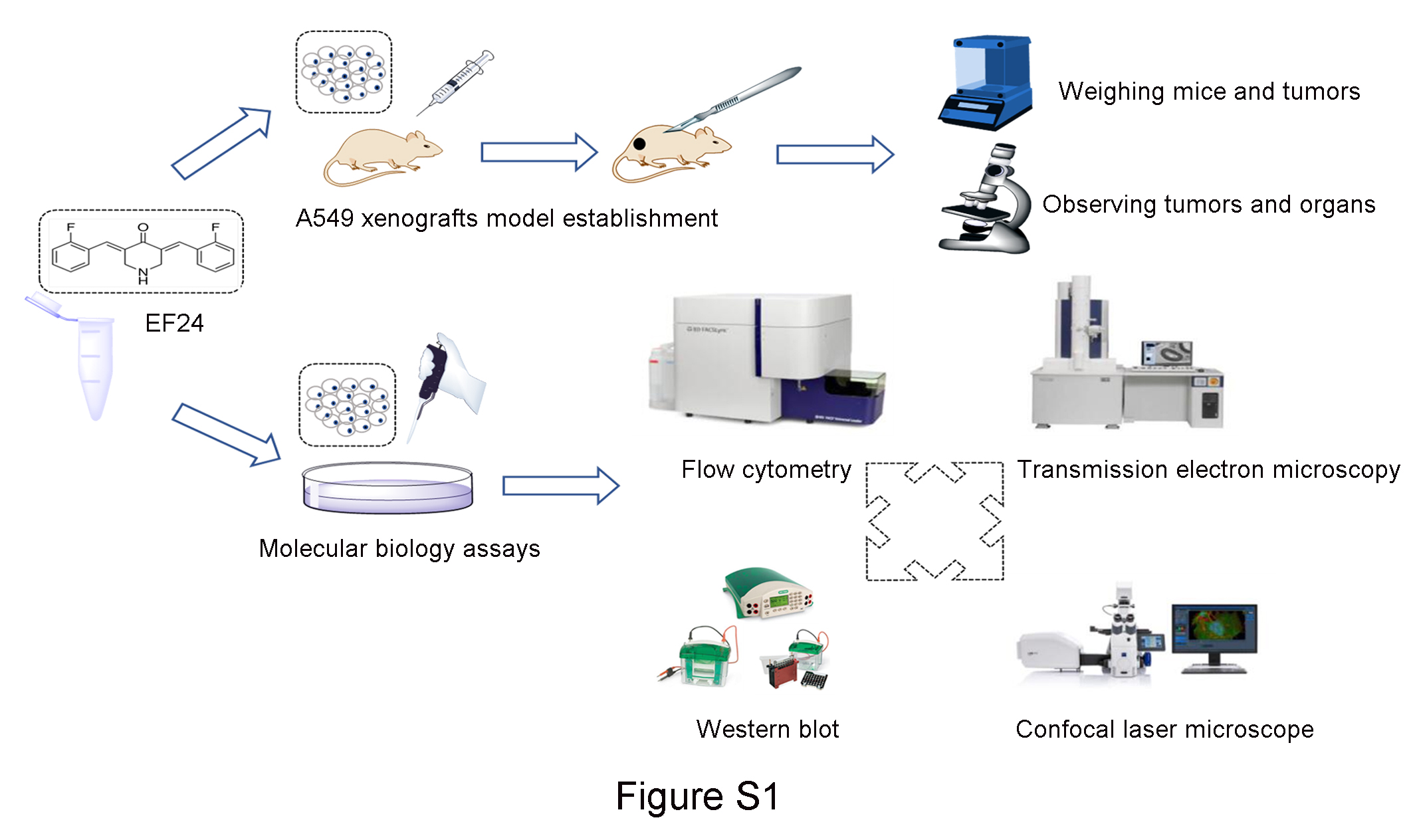

Supplement: Supplementary file 2 — Additional file 2: Fig. S2. Schematic Illustration of the main research methodology. [file 12935_2021_2240_MOESM2_ESM.jpg]
